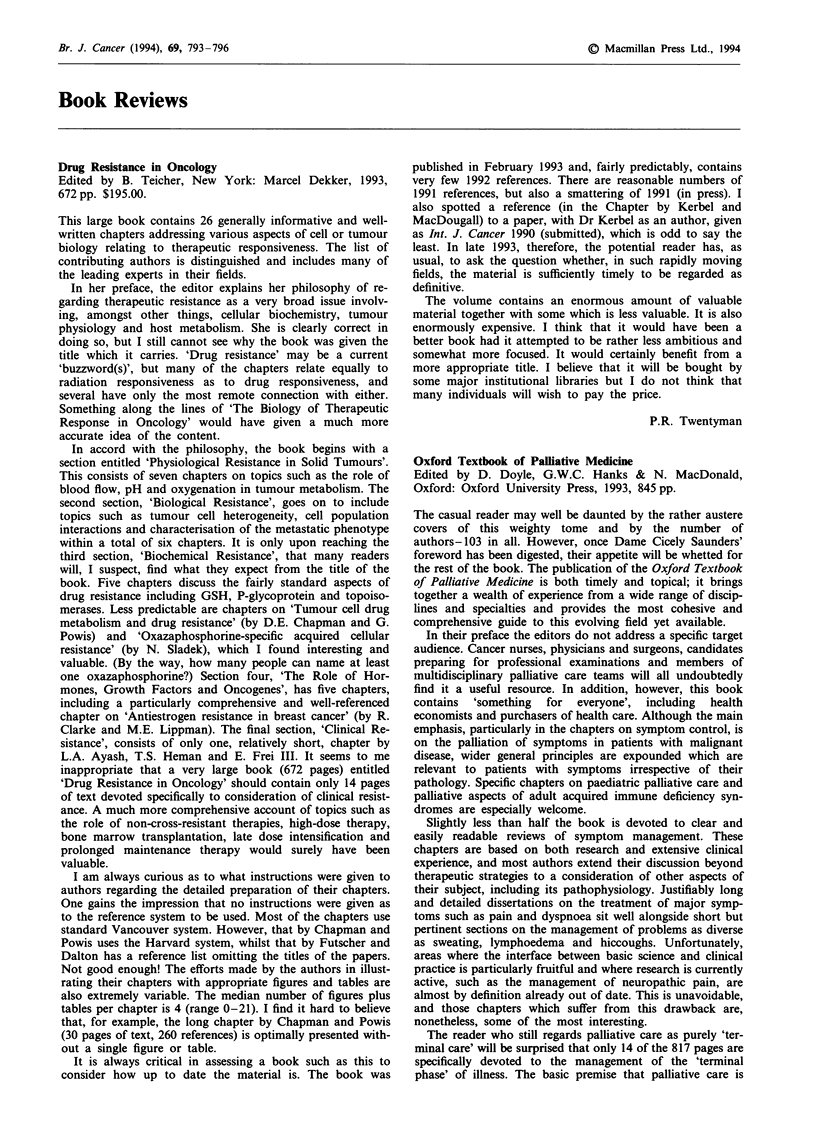# Drug Resistance in Oncology

**Published:** 1994-04

**Authors:** P.R. Twentyman


### Book Reviews

**Drug Resistance in Oncology**
Edited by B. Teicher, New York: Marcel Dekker, 1993, 672 pp. $195.00.

This large book contains 26 generally informative and well-written chapters addressing various aspects of cell or tumour biology relating to therapeutic responsiveness. The list of contributing authors is distinguished and includes many of the leading experts in their fields.

In her preface, the editor explains her philosophy of regarding therapeutic resistance as a very broad issue involving, amongst other things, cellular biochemistry, tumour physiology and host metabolism. She is clearly correct in doing so, but I still cannot see why the book was given the title which it carries. 'Drug resistance' may be a current 'buzzword(s)', but many of the chapters relate equally to radiation responsiveness as to drug responsiveness, and several have only the most remote connection with either. Something along the lines of 'The Biology of Therapeutic Response in Oncology' would have given a much more accurate idea of the content.

In accord with the philosophy, the book begins with a section entitled 'Physiological Resistance in Solid Tumours'. This consists of seven chapters on topics such as the role of blood flow, pH and oxygenation in tumour metabolism. The second section, 'Biological Resistance', goes on to include topics such as tumour cell heterogeneity, cell population interactions and characterisation of the metastatic phenotype within a total of six chapters. It is only upon reaching the third section, 'Biochemical Resistance', that many readers will, I suspect, find what they expect from the title of the book. Five chapters discuss the fairly standard aspects of drug resistance including GSH, P-glycoprotein and topoisomerases. Less predictable are chapters on 'Tumour cell drug metabolism and drug resistance' (by D.E. Chapman and G. Powis) and 'Oxazaphosphorine-specific acquired cellular resistance' (by N. Sladek), which I found interesting and valuable. (By the way, how many people can name at least one oxazaphosphorine?) Section four, 'The Role of Hormones, Growth Factors and Oncogenes', has five chapters, including a particularly comprehensive and well-referenced chapter on 'Antiestrogen resistance in breast cancer' (by R. Clarke and M.E. Lippman). The final section, 'Clinical Resistance', consists of only one, relatively short, chapter by L.A. Ayash, T.S. Heman and E. Frei III. It seems to me inappropriate that a very large book (672 pages) entitled 'Drug Resistance in Oncology' should contain only 14 pages of text devoted specifically to consideration of clinical resistance. A much more comprehensive account of topics such as the role of non-cross-resistant therapies, high-dose therapy, bone marrow transplantation, late dose intensification and prolonged maintenance therapy would surely have been valuable.

I am always curious as to what instructions were given to authors regarding the detailed preparation of their chapters. One gains the impression that no instructions were given as to the reference system to be used. Most of the chapters use standard Vancouver system. However, that by Chapman and Powis uses the Harvard system, whilst that by Futscher and Dalton has a reference list omitting the titles of the papers. Not good enough! The efforts made by the authors in illustrating their chapters with appropriate figures and tables are also extremely variable. The median number of figures plus tables per chapter is 4 (range 0–21). I find it hard to believe that, for example, the long chapter by Chapman and Powis (30 pages of text, 260 references) is optimally presented without a single figure or table.

It is always critical in assessing a book such as this to consider how up to date the material is. The book was published in February 1993 and, fairly predictably, contains very few 1992 references. There are reasonable numbers of 1991 references, but also a smattering of 1991 (in press). I also spotted a reference (in the Chapter by Kerbel and MacDougall) to a paper, with Dr Kerbel as an author, given as *Int. J. Cancer* 1990 (submitted), which is odd to say the least. In late 1993, therefore, the potential reader has, as usual, to ask the question whether, in such rapidly moving fields, the material is sufficiently timely to be regarded as definitive.

The volume contains an enormous amount of valuable material together with some which is less valuable. It is also enormously expensive. I think that it would have been a better book had it attempted to be rather less ambitious and somewhat more focused. It would certainly benefit from a more appropriate title. I believe that it will be bought by some major institutional libraries but I do not think that many individuals will wish to pay the price.

P.R. Twentyman